# β-Glucosidase and β-Galactosidase-Mediated Transglycosylation of Steviol Glycosides Utilizing Industrial Byproducts

**DOI:** 10.3389/fbioe.2021.685099

**Published:** 2021-06-09

**Authors:** Anastasia Zerva, Koar Chorozian, Anastasia S. Kritikou, Nikolaos S. Thomaidis, Evangelos Topakas

**Affiliations:** ^1^Biotechnology Laboratory, School of Chemical Engineering, National Technical University of Athens, Athens, Greece; ^2^Laboratory of Analytical Chemistry, Department of Chemistry, National and Kapodistrian University of Athens, Athens, Greece

**Keywords:** stevioside, rebaudioside A, transglycosylation, β-glucosidase, β-galactosidase

## Abstract

*Stevia rebaudiana* Bertoni is a plant cultivated worldwide due to its use as a sweetener. The sweet taste of stevia is attributed to its numerous steviol glycosides, however, their use is still limited, due to their bitter aftertaste. The transglycosylation of steviol glycosides, aiming at the improvement of their taste, has been reported for many enzymes, however, glycosyl hydrolases are not extensively studied in this respect. In the present study, a β-glucosidase, *Mt*Bgl3a, and a β-galactosidase, *Tt*bGal1, have been applied in the transglycosylation of two steviol glycosides, stevioside and rebaudioside A. The maximum conversion yields were 34.6 and 33.1% for stevioside, while 25.6 and 37.6% were obtained for rebaudioside A conversion by *Mt*Bgl3a and *Tt*bGal1, respectively. Low-cost industrial byproducts were employed as sugar donors, such as cellulose hydrolyzate and acid whey for *Tt*bGal1- and *Mt*Bgl3a- mediated bioconversion, respectively. LC-HRMS analysis identified the formation of mono- and di- glycosylated products from stevioside and rebaudioside A. Overall, the results of the present work indicate that both biocatalysts can be exploited for the design of a cost-effective process for the modification of steviol glycosides.

## Introduction

*Stevia rebaudiana* Bertoni is a plant native to south America, cultivated worldwide due to its use as a low-caloric sweetener. Stevia stems and leaves are rich in diterpenoid glycosides, mainly stevioside and rebaudioside A (rebA), but many more minor glycosides have been isolated. The chemical structure of stevia glycosides includes the diterpenoid steviol, glycosylated through an ester bond at the C13 or C19 with one or more glucose moieties, connected with β-1,2 or β-1,3 glycosidic bond (Nguyen et al., 2019).

Considering the growing demand for natural, unprocessed foods, it is evident that natural sweeteners are also very popular, especially for individuals suffering from diabetes or obesity. However, steviol glycoside-containing products are not so well received by a large number of consumers, due to their lingering bitter aftertaste. Although the exact correlation between glycoside structure and taste has not been elucidated, a study by Singla and Jaitak (2016) has shown that the structure of the glycoside determines their recognition by the bitter taste receptors of the human tongue. In contrast to stevioside, rebaudioside A, due to an additional sugar moiety, is unable to interact with the receptor, and therefore has less lingering bitter taste than stevioside. Therefore, significant research effort has been focused on the addition of glycosyl moieties in steviol glycosides, aiming at the improvement of their organoleptic properties. Cyclodextrin glucanotransferases (CGTases, 1,4-α-d-glucan: 1,4-α-d-glucopyranosyltransferase, cyclizing, EC 2.4.1.19) are the most common enzymes used for steviol glycosides transglycosylation, introducing α-1,4 glycosidic bonds with very high yields, exceeding 70% (Li et al., 2013; Yu et al., 2015; Zhang et al., 2015). However, the hydrolysis of α- 1,4 bonds by human amylases can increase the caloric value of the modified steviosides, resulting in a final product with low appeal to target consumer groups, e.g., diabetics (Gerwig et al., 2016). Nonetheless, the increase is expected to be very low, or even negligible, considering the low dose of steviol glucosides required to obtain the desired level of sweetness.

Another promising enzyme group regarding the modification of steviol glycosides is glucansucrases (EC 2.1.4.-). Glucansucrases are found only in lactic acid bacteria, and they are able to introduce α- 1,6 and α- 1,3 glycosidic bonds to steviol glycosides, with high yields (Gerwig et al., 2017; Devlamynck et al., 2019), often higher than 90% (Ko et al., 2016). The advantages of their use also include the use of sucrose as a low-cost sugar donor, as well as the formation of glycosidic bonds that are not hydrolyzed by human digestive enzymes.

The modification of stevioside can also be achieved by the action of the native enzyme present in stevia plant, UDP-glucosyltransferase (EC 2.4.1.-). These enzymes introduce β-glycosidic bonds to the steviol glycosides, which makes them ideal candidates for their biocatalytic modification, since these bonds are not hydrolyzed in the human body. However, the need for UDP- activated donors greatly increases the cost of the process. Nonetheless, a few interesting efforts were described, to develop a biocatalytic system for the *in situ* regeneration of UDP donors (Wang et al., 2016; Chen et al., 2018) employing sucrose synthase.

Regarding hydrolases, although they have been used extensively in the literature for various transglycosylation reactions, studies reporting the transglycosylation of steviol glycosides are sparse. β-Glucosidases and β-galactosidases have been used mainly for the hydrolysis of stevioside, aiming at the production of less glucosylated products (Ko et al., 2012, 2013; Nguyen et al., 2014; Chen et al., 2016), while a few studies report the transgalactosylation by β-galactosidases (Kitahata et al., 1989). According to the literature, β-galactosidases usually transgalactosylate their acceptor substrates (Irazoqui et al., 2009). Transfructosylation has also been described (Ishikawa et al., 1990).

In the present study, two novel glycosyl hydrolases were employed for the transglycosylation of stevioside and rebA. *Mt*Bgl3a is a β-glucosidase, acting on cellobiose, with previously documented transglycosylation activity (Karnaouri et al., 2013), while *Tt*bGal1 is a β-galactosidase, also shown to be able to transglycosylate its donor lactose to galactooligosaccharides with up to 4 sugar moieties (Zerva et al., 2021). Both enzymes are thermostable, since the respective genes were isolated from thermophile fungi (*Tt*bGal1 from *Thielavia terrestris* and *Mt*Bgl3a from *Thermothelomyces thermophila*, respectively). Using lactose or cellobiose as the sugar donor, stevioside and rebA were modified to more glycosylated products. Low-cost industrial byproducts were tested as sugar donors, for the cost-effective modification of steviol glycosides. Different forms of glycosylated products were identified using LC-HRMS.

## Materials and Methods

### Enzymes and Chemicals

Cellobiohydrolase I (CBH1) and endoglucanase 7 (EG7) were purchased from Megazyme (Bray, Co. Wicklow, Ireland). Avicel was purchased from Merck KGaA, Darmstadt, Germany. Stevioside (>85%) and Rebaudioside A (>98%) were purchased from TCI America (Boston, MA, United States). All other chemicals were purchased from Merck KGaA, Darmstadt, Germany, and they were of the highest purity available.

### Acid Whey

The acid whey that was used for the enzymatic production of GOS was provided by Delta Foods SA and it was obtained from production processes of Greek strained fat-free yogurt. The composition was as follows; total protein, 0.2% (w/w); fat, 0.07% (w/w); TSS 4.95% (w/w); ash, 0.70% (w/w). The initial lactose concentration was 3.4% (w/v), the initial monosaccharides (glucose and galactose) concentration was 0.79% (w/v) and the pH of the whey was 4.3 ± 0.25. In order to concentrate the lactose contained in the whey, a Hei-VAP value digital rotary evaporator (Heidolph Instruments GmbH & CO. KG) was used. The temperature of the water bath and the rotation speed were set to 52°C and 120 rpm respectively. After concentration, the final lactose concentration was 9.3% (w/v).

### Production and Purification of Recombinant *Tt*bGal1 and *Mt*Bgl3a

*Tt*bGal1 was produced and purified as a heterologous enzyme expressed in *Pichia pastoris*, as described previously (Zerva et al., 2021). *Mt*Bgl3a was similarly produced in *P. pastoris* host, as described previously (Karnaouri et al., 2013). The recombinant enzymes were purified from the culture filtrate with the use of an immobilized metal-ion affinity chromatography (IMAC) column (Talon, Clontech; 1.0 cm i.d., 15 cm length), as described previously (Karnaouri et al., 2013; Zerva et al., 2021). Protein quantification was performed, as previously described (Bradford, 1976).

### Determination of Enzyme Activity

*Tt*bGal1 activity was routinely assayed with o-NPhG as the substrate (0.5 mM) in 50 mM phosphate – citrate buffer pH 4 at 40°C. *Mt*Bgl3a activity was assayed with 1 mM p-β-NPhG (p-NPh-β-D-glucopyranoside) in 0.1 M citrate-phosphate buffer pH 5.0 for 10 min at 50°C. For both enzyme assays, the reaction was stopped after 10 min with 1 M Na_2_CO_3_, and the absorbance at 410 nm was performed in a Spectra Max 250 microplate reader (Molecular Devices, CA, United States). 1 Unit of enzymatic activity was defined as the amount of enzyme that hydrolyzes 1 μmol of substrate per min.

### Transglycosylation of Steviol Glycosides

The transglycosylation of stevioside and RebA was performed in 1 mL reactions, in an Eppendorf thermomixer, under vigorous stirring. *Tt*bGal1-mediated bioconversion, was routinely performed with 100 g L^–1^ lactose as the donor substrate, 5 g L^–1^ of either stevioside or RebA, 0.5 U mL^–1^ *Tt*bGal1, in phosphate-citrate buffer pH 4.5, in 45°C. *Mt*Bgl3a -mediated bioconversion was routinely performed with 50 g L^–1^ cellobiose as the donor substrate, 5 g L^–1^ of either stevioside or RebA, 64 μg mL^–1^ (6.25 U mL^–1^) *Mt*Bgl3a, in phosphate-citrate buffer pH 5, in 45°C. Sodium azide was also added to avoid microbial contamination in prolonged reactions, at a final concentration of 0.02% (w/v).

The acid whey preparation was also utilized for transgalactosylation reactions, with lactose concentration of 9.3% (w/v). *Tt*bGal1 was added to 1 mL of acid whey, with an activity of 0.5 U mL^–1^, together with 5 g L^–1^ of either stevioside or RebA. The mixtures were incubated at 45°C in a shaking thermomixer.

*Mt*Bgl3a was also used for the transglycosylation of stevioside and RebA, with hydrolyzed microcrystalline cellulose (Avicel) as donor substrate. Avicel (100 g L^–1^) was hydrolyzed to cello-oligosaccharides by CBHI (4.5 mg g^–1^) and EG7 (2.7 mg g^–1^), at 1 mL reactions for 16 h in 45 °C, pH 5.5 under agitation (950 rpm). Then, *Mt*Bgl3a was added at a final concentration of 64 μg mL^–1^, and stevioside or RebA at a final concentration of 5 g L^–1^, and the reaction was incubated for a further 24 h.

For all reactions, 0.5 mL aliquots were removed from the reaction mixture at selected time intervals. The aliquots were immediately boiled for 10 min, filtered using 0.22 μm PVDFL filters and diluted suitably prior to analysis.

### Chromatographic Analysis of Transglycosylation Products

An LC-20AD HPLC system (Shimadzu) was employed for the analysis of the reaction products using a C18 CC 250/4.6 Nucleosil 100-5 column (Macherey-Nagel). The mobile phase consisted of 70% (v/v) phosphate buffer 10 mM pH 2.6 and 30% (v/v) acetonitrile. After sample injection (25 μL), chromatographic separation of steviol glycosides was performed with isocratic elution, at 1 mL min^–1^, while monitoring was performed at 210 nm using a UV–vis ProStar 335 Diode Array detector (Agilent Technologies). Stevioside and rebA concentrations were determined against a suitable calibration curve.

The mono-, di- and oligosaccharides contained in the aliquots were separated with an HPLC system, using a Microsorb-MV 100 NH_2_ (250 mm × 4.6 mm) column equipped with a Polaris 5 NH_2_ MetaGuard guard column (Agilent Technologies Sales & Services GmbH & Co. KG, Santa Clara, CA, United States). The eluent used for the separation was 75% (v/v) acetonitrile in water and the flow rate was set to 1.0 mL min^–1^. The sugars were detected using a Shimadzu RID-10A refractive index detector (Hewllet-Packard Company, Palo Alto, CA, United States). The identification and quantification of the sugars were performed using galactose, lactose and raffinose as standard compounds for mono-, di- and oligosaccharides, respectively (Fischer and Kleinschmidt, 2015).

### LC-HRMS Analysis of Transglycosylation Products

#### LC-HRMS Instrumentation

In order to tentatively identify the steviol glycosides produced during the reactions, accurate data were obtained using Liquid Chromatography coupled to High Resolution Mass Spectrometry (LC-HRMS). An Ultrahigh-Performance Liquid Chromatography (UHPLC) system with a HPG-3400 pump (Dionex Ultimate 3000 RSLC, Thermo Fischer Scientific, Dreieich, Germany) interfaced to a Quadrupole Time-of-Flight (QTOF) mass spectrometer (Maxis Impact, Bruker Daltonics, Bremen, Germany) was employed.

A Hydrophilic Interaction Liquid Chromatography (HILIC) system was applied for the chromatographic separation of the examined transglycosylation products. An ACQUITY BEH Amide chromatographic column (2.1 × 100 mm, 1.7 μm) from Waters (Dublin, Ireland) was used, preceded by a guard column of the same material, thermostated at 40°C. The mobile phases used for the analysis consisted of deionized water (solvent A) and acetonitrile/water 95/5 (solvent B) both containing 1 mM ammonium formate and 0.01% formic acid. A gradient elution program was performed, started with 100% B (0–2 min), decreasing to 5% within 10 min (t:12 min) and kept constant for the following 5 min (t:17 min). Restorage of initial conditions was carried out within 0.1 min and a post-run of 8 min was programmed to re- equilibrate the column between analyses, resulting to a total analysis time of 25 min. The constant flow rate was set at 0.2 mL min^–1^ while the injection volume was 5 μL. Samples from enzymatic reactions were subjected to serial dilution 100-fold in ACN/water (80:20) prior to analysis.

HRMS analysis was achieved by a QTOF system equipped with an Electrospray Ionization source (ESI), operating in negative ionization mode, with the following ionization parameters: capillary voltage 2500 V (PI) and 3500 (NI); end plate offset, 500 V; nebulizer pressure 2 bar; drying gas 8 L min^–1^ and dry temperature 200°C. QTOF MS system was operated in broadband collision- induced dissociation (bbCID) acquisition mode, providing MS and MS/MS spectra within the same injection using two different collision energies. At low collision energy (4 eV), MS spectra were acquired and at high collision energy (25 eV), fragmentation is taking place at the collision cell resulting in MS/MS spectra. Recording of full scan mass spectra for each sample was elaborated in a mass range of 50–1000 m/z, setting a scan rate of 2 Hz. For the accuracy of the obtained mass spectra, MS external calibration was performed using as calibrant 10 mM sodium formate diluted in a mixture of water/isopropanol (1:1). The theoretical exact masses of calibration ions with formulas Na (NaCOOH) 1-14 in the range of 40-1500 Da were used for calibration. Additional internal calibration was also performed by a calibrant injection at the beginning of each chromatogram within 0.1-0.25 min.

#### Data Treatment

Data acquisition was carried out with the HyStar and Compass software (Bruker Daltonics, Bremen, Germany). Mass spectra interpretation was performed by Data Analysis 4.4 software packages (Bruker Daltonics, Bremen, Germany). Since no reference standards were available for all the transglycosylated products, a suspect screening approach was applied for compounds’ detection and identification. An in-house suspect database was built including the all the possible transglycosylated products. Molecular formulas of the suspect analytes, pseudomolecular ions [M-H]^–^ and possible adduct formations ([M + HCOOH-H]^–^ etc.) occurring during the ionization processing were included in the database.

Full-scan chromatogram of each injected sample was screened for all the suspect products. Exploiting Data Analysis software, Extracted Ion Chromatograms (EIC) were created for all detected mass features and the obtained peaks were evaluated according to specific detection and identification criteria (peak area, intensity, mass accuracy, isotopic fitting and MS/MS fragmentation). Based on our previous study, for the accurate evaluation of the resulting chromatographic data, strict chromatographic parameters were set: peak area threshold was set >800, intensity threshold >200 and a signal to noise ratio at 3 (Kalogiouri et al., 2016). For identification purposes, crucial MS parameters were appointed. Mass accuracy window was set at 5 mDa for both precursor and detected fragment ions, mSigma values (comparing the theoretical and measured isotopic profiles) were exploited for isotopic fitting evaluation which should not be more than 50. Bruker Software Tools included in the above mentioned softwares (Compass Isotope Pattern and SmartFormula Manually) were used for confirmation purposes. *In silico* fragmentation tools like Metfrag and spectral libraries such as MassBank were also used for the estimation and interpretation of MS/MS fragments (Wolf et al., 2010).

### Statistical Analysis

Data analysis was performed with SigmaPlot v12.5 software (Systat Software, Inc., San Jose, CA, United States). Error bars represent the standard deviation of the mean value.

## Results

### Utilization of Defined Sugar Donors

#### *Tt*bGal1-Mediated Transglycosylation of Steviol Glycosides

**FIGURE 1:**
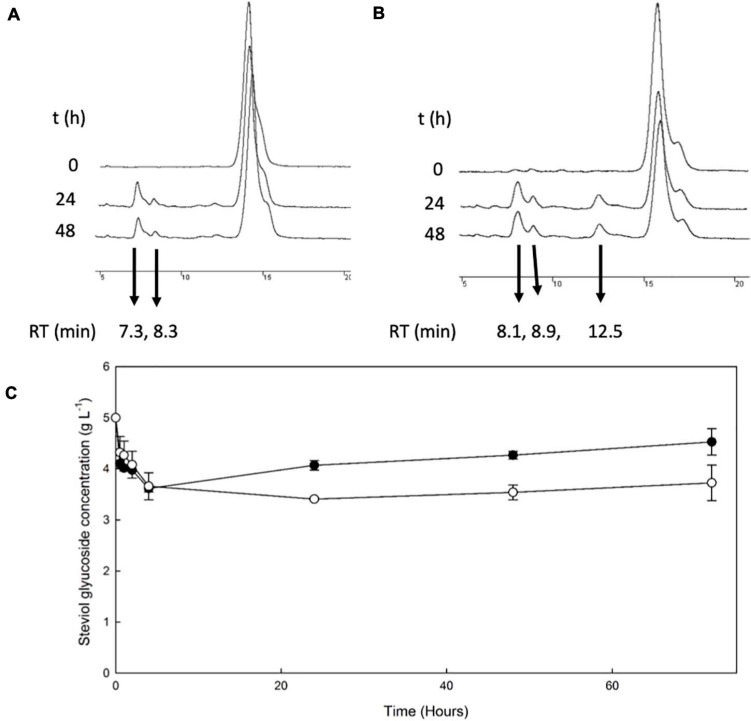
Transglycosylation of steviol glycosides by *Tt*bGal1. HPLC chromatograms of the reaction mixture of *Tt*bGal1 with stevioside **(A)** and RebA **(B)**, with lactose as the donor **(C)** *Tt*bGal1-mediated bioconversion of stevioside (*black squares*) and RebA (*white circles*).

The β-galactosidase *Tt*bGal1 was employed for the transglycosylation of stevioside and RebA, using lactose as donor. HPLC chromatograms of selected time points during the reaction are shown in Figures 1A,B. During the reaction with stevioside as acceptor, two extra peaks were visible, corresponding to shorter retention times (7.3 and 8.3 min), while for RebA three extra peaks were visible (8.07, 8.9, and 12.55). The conversion of stevioside and RebA was monitored during the course of the reaction, and the results are shown in Figure 1C.

The conversion of both stevioside and RebA started almost immediately, and the lowest concentration was measured after 4 h of reaction in the case of stevioside, and after 24 h of reaction for RebA. After that, the concentration of both glycosides started to rise slowly, indicating a certain degree of hydrolysis of the transglycosylated products. The maximum conversion of stevioside and RebA was 27.7 ± 1.4% and 31.8 ± 0.5% respectively.

**FIGURE 2:**
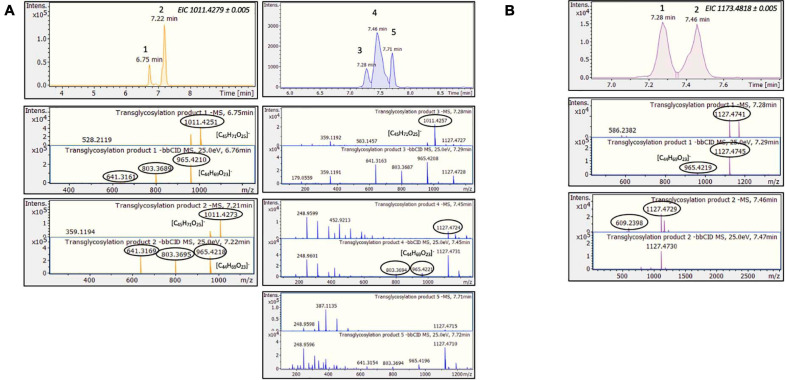
*Tt*bGal1-mediated transglycosylation of steviol glycosides. EICs, MS and MS/MS spectra of the detected transglycosylated products provided by HILIC-HRMS using as acceptor **(A)** stevioside **(B)** RebA.

Considering the above HRMS-based workflow, during both transglycosylation reactions, different glycosylated products were detected and identified. The majority of the identified products were detected in two ionized forms ([M-H]^–^ and [M + HCOOH-H]^–^), enhancing the identification points. The most abundant precursor ion was found to be the formate adduct [M + HCOOH-H]^–^. During the transglycosylation reaction using stevioside as acceptor, two different chromatographic peaks were detected in 6.75 and 7.22 min, respectively, corresponding to two different mono-glycosylated products with the same molecular formula (C_44_H_70_O_23_). Three di-glycosylated products (C_50_H_80_O_28_) were also detected, corresponding to retention times 7.28, 7.46, and 7.71 min. Respectively, during conversion of RebA two chromatographic peaks were detected, corresponding to the formation of mono- glycosylated products with molecular formula (C_50_H_80_O_28_). Chromatograms and annotated MS and MS/MS spectra - corresponding to the detected products are provided in Figures 2A,B. The obtained mass spectra presented high mass accuracy (less than 5 mDa) and satisfactory isotopic patterns (less than 50 mSigma) in both cases. Using fragmentation tools and spectral libraries reported above, characteristic fragments of the reported products were identified for the most abundant precursor ion, reaching the identification of molecular formulas (Figures 2A,B).

During RebA transglycosylation, the concentration of donor lactose was also monitored, and the results are shown in Supplementary Figure 1A. From these results it is obvious that *Tt*bGal1 is active during the whole reaction time, since the consumption of lactose is continuous up to 72 h. As shown in Supplementary Figure 1A, more than 60% of lactose is consumed in the 72 h of the reaction, but also monosaccharides (glucose and galactose) are produced from the simultaneous lactose hydrolysis, up to 33.3 ± 2.7 g L^–1^. Moreover, *Tt*bGal1 is able to transglycosylate lactose, and small quantities of galactooligosaccharides (GOS) are produced (Supplementary Figure 2A). Based on LC-HRMS analysis, the results reported in Supplementary Figures 3, 4 indicate the production of di-, tri- and tetrasaccharides in most reactions.

**FIGURE 3:**
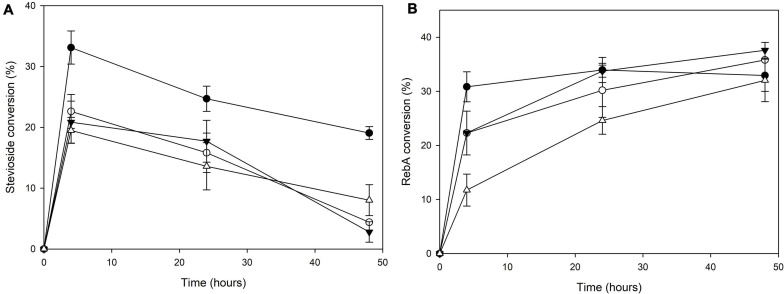
Conversion of different concentrations of **(A)** stevioside and **(B)** RebA by *Tt*bGal1. *Black circles*: 2.5 g L^–1^ glycoside, *white circles*: 5 g L^–1^ glycoside, *black inverted triangles*: 7.5 g L^–1^ glycoside, *white triangles*: 10 g L^–1^ glycoside.

The transglycosylation reaction was also tested in different concentrations of steviol glycosides, and the results are shown in Figure 3. For stevioside transglycosylation, the optimum conversion (33.1 ± 2.7%) was observed again for 4 h reaction, but in lower starting concentration, 2.5 g L^–1^, while the maximum concentration of consumed stevioside was observed in 10 g L^–1^ after 4 h reaction (2.0 ± 0.2 g L^–1^). For RebA transglycosylation, the optimum conversion (37.6 ± 1.5%) was observed for 7.5 g L^–1^, after 48 h of reaction, but the maximum concentration of consumed RebA was observed in 10 g L^–1^ (3.2 ± 0.6 g L^–1^).

#### *Mt*Bgl3a -Mediated Transglycosylation of Steviol Glycosides

**FIGURE 4:**
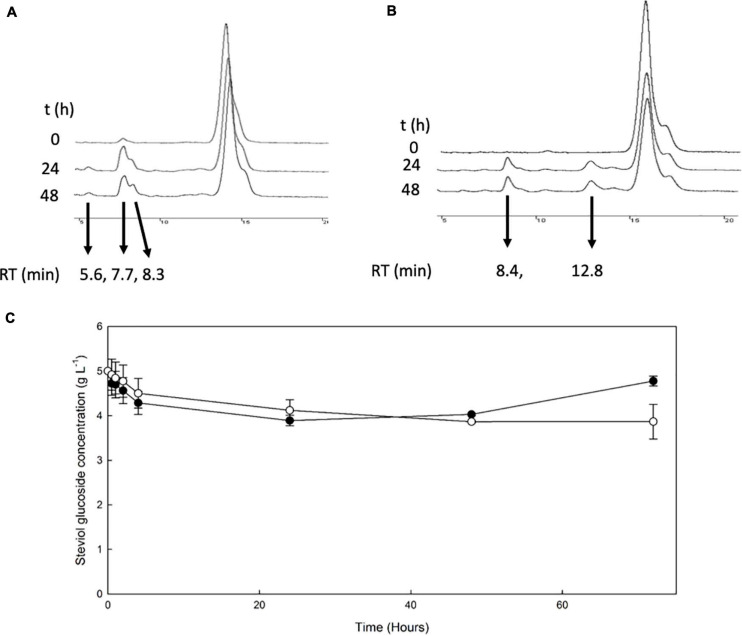
Transglycosylation of steviol glycosides by *Mt*Bgl3a. HPLC chromatograms of the reaction mixture of *Mt*Bgl3a with stevioside **(A)** and RebA **(B)**, with cellobiose as the donor. **(C)** *Mt*Bgl3a -mediated bioconversion of stevioside (*black squares*) and RebA (*white circles*).

The β-glucosidase *Mt*Bgl3a was also employed for the transglycosylation of stevioside and RebA, using cellobiose as donor. HPLC chromatograms of selected time points during the reaction are shown in Figures 4A,B.

**FIGURE 5:**
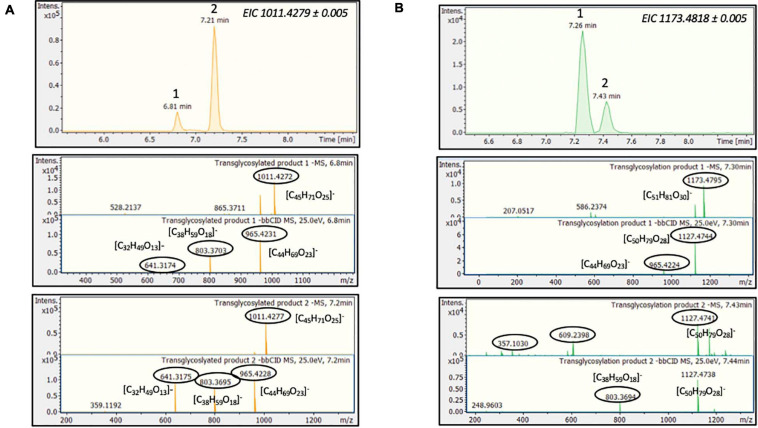
*MtBGL3a*-mediated transglycosylation of steviol glycosides. EICs, MS and MS/MS spectra of the detected transglycosylated products provided by HILIC-HRMS using as acceptor **(A)** stevioside **(B)** RebA.

During the reaction with stevioside as the acceptor, three extra peaks appeared, corresponding to shorter retention times (5.6, 7.7, and 8.3 min), while for RebA two extra peaks appeared (8.4 and 12.8 min). The conversion of stevioside and RebA was monitored during the course of the reaction, and the results are shown in Figure 4C. The conversion of both stevioside and RebA started almost immediately, and the lowest concentration was measured after 24 h of reaction in the case of stevioside, after which the concentration of stevioside started to rise, indicating a certain degree of hydrolysis of the transglycosylated products. In the case of RebA transglycosylation, the conversion of RebA reached a plateau after 48 h, and no significant change was observed after that point. The maximum conversion of stevioside and RebA was 22.2 ± 2.4% and 22.7 ± 0.5% respectively. The results were confirmed by HILIC-HRMS analysis in both transglycosylation reactions, as two chromatographic peaks of mono-glycosylated products were observed in each case. Specifically, in the case of stevioside transglycosylation, two peaks were detected in 6.81 and 7.22 min corresponding to C_44_H_70_O_23_ glycoside, while in the case of RebA transglycosylation peaks were detected in 7.26 and 7.43 min corresponding to C_50_H_80_O_28_, respectively (Figures 5A,B).

In the case of RebA transglycosylation reaction, the concentration of donor cellobiose was also monitored, and the results are shown in Supplementary Figure 1B. From these results, it is obvious that *Mt*Bgl3a is active during the whole reaction time, since the consumption of cellobiose is continuous up to 72 h. As shown in Supplementary Figure 1B, more than 50% of cellobiose is consumed in the 72 h of the reaction, but also monosaccharides (glucose) are produced from the simultaneous cellobiose hydrolysis, up to 10.2 ± 0.8 g L^–1^. Moreover, *Mt*Bgl3a is able to transglycosylate cellobiose, and small quantities of cellooligosaccharides (COS) are produced (Supplementary Figure 2B). The results confirmed by LC-HRMS data, as cellobiose (C_12_H_22_O_11_), cellotriose (C_18_H_32_O_16_) and cellotetraose (C_24_H_42_O_21_) were detected in all the related reactions (Supplementary Figures 3, 4).

**FIGURE 6:**
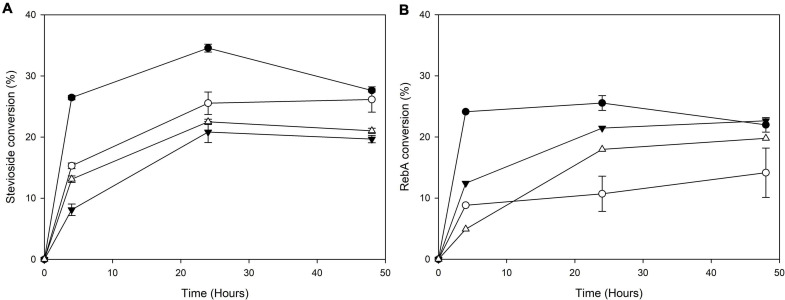
Conversion of different concentrations of **(A)** stevioside and **(B)** RebA by *Mt*Bgl3a. *Black circles*: 2.5 g L^–1^ glycoside, *white circles*: 5 g L^–1^ glycoside, *black inverted triangles*: 7.5 g L^–1^ glycoside, *white triangles*: 10 g L^–1^ glycoside.

The transglycosylation reaction was also tested in different concentrations of steviol glycosides, and the results are shown in Figure 6.

For stevioside transglycosylation, the optimum conversion (34.6 ± 0.6%) was observed again for 24 h reaction, but in lower starting concentration, 2.5 g L^–1^, while the maximum concentration of consumed stevioside was observed in 10 g L^–1^ after 24 h reaction (2.3 ± 0.04 g L^–1^). For RebA transglycosylation, the optimum conversion (25.6 ± 1.2%) was observed for 2.5 g L^–1^, after 24 h of reaction, but the maximum concentration of consumed rebA was observed in 7.5 g L^–1^ after 48 h reaction (2.3 ± 0.4 g L^–1^).

### Utilization of Acid Whey Wastewater and Microcrystalline Cellulose

#### *Tt*bGal1-Mediated Transglycosylation of Steviol Glycosides

**FIGURE 7:**
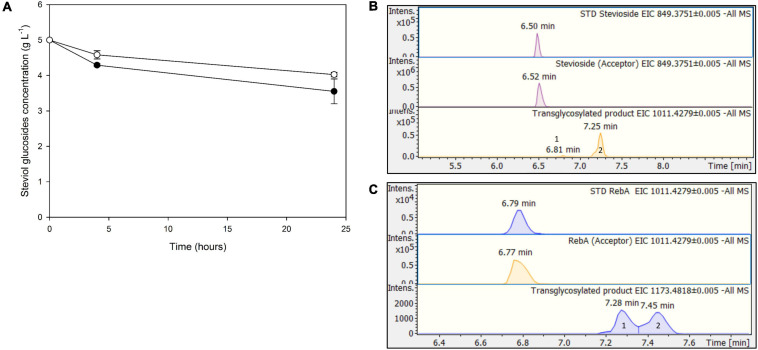
**(A)** Transglycosylation of steviol glycosides by *Tt*bGal1 with concentrated acid whey as the donor. *Black circles*: stevioside, *white circles*: RebA **(B)**, EICs of the detected transglycosylated products provided by HILIC-HRMS using stevioside **(B)** or RebA **(C)** as acceptor.

The next step was to use low-cost industrial byproducts as the source of donor sugars for the transglycosylation of steviol glycosides. In the case of *Tt*bGal1, concentrated acid whey wastewater, rich in residual lactose, was used. In this case, hydrolysis of the transglycosylation products was not observed, since the concentration of steviol glycosides decreased up to 24 h (Figure 7A). The maximum conversion observed for stevioside was 28.9 ± 7%, while for RebA the maximum conversion was 19.5 ± 1.2%. Based on HILIC-HRMS data, the transglycosylation of steviol glycosides was achieved, detecting two different mono- glycosylated products in each reaction. The results are shown in Figures 7B,C. The mono-glycosylated products are in compliance with the detected products shown in Figures 2A,B, as the products present similar retention times, MS and MS/MS patterns meeting the evaluation criteria reported previously.

#### *Mt*Bgl3a -Mediated Transglycosylation of Steviol Glycosides

**FIGURE 8:**
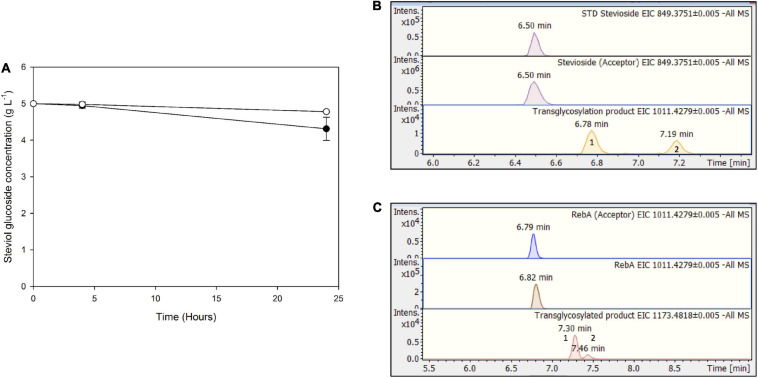
**(A)** Transglycosylation of steviol glycosides by *Mt*Bgl3a with hydrolyzed microcrystalline cellulose as the donor. *Black circles*: stevioside, *white circles*: RebA. EICs of the detected transglycosylated products provided by HILIC-HRMS using as acceptor **(B)** stevioside **(C)** RebA.

For *Mt*bgl3a-mediated bioconversion of steviol glycosides, cellulose was chosen as a substrate, since it is available as a byproduct from most agroindustrial activities, as part of lignocellulosic biomass. In this case, significantly lower conversion yields were obtained compared to the use of pure cellobiose as donor, up to 13.8 ± 3.4 for stevioside, and 4.4 ± 0.5% for RebA (Figure 8A). This observation is probably due to the low yield of cellulose hydrolysis obtained from cellulases (Karnaouri et al., 2019), resulting to low starting concentrations of donor cellobiose, but also to variable specificity of β-glucosidases toward the hydrolysis of COS, produced during the enzymatic degradation of cellulose, compared to cellobiose (Guo et al., 2016). According to HILIC-HRMS analysis, during the processes using cellulose as a substrate, two different chromatographic peaks were also detected, corresponding to mono-glycosylated products (Figures 8B,C). Mono-glycosylated products detected using cellulose as a substrate indicate the effectiveness of low-cost industrial byproducts as donors, as the same mono - glycosylated products in higher abundance were detected in the case of pure cellobiose as a donor.

## Discussion

In the present work, two glycosyl hydrolases, a β-galactosidase *Tt*bGal1, and a β-glucosidase *Mt*Bgl3a, were employed for the transglycosylation of steviol glycosides, stevioside and rebA. Both enzymes were selected for this particular biocatalytic reaction, due to their ability to perform transglycosylation reactions, inserting β-glycosyl residues to acceptor substrates. As shown previously, increasing degree of β-glycosylation of steviosides correlates with increasing sweetness (Hellfritsch et al., 2012). More importantly, both tested enzymes are valuable catalysts in transglycosylation reactions due to their thermophile nature (Karnaouri et al., 2013; Zerva et al., 2021). Their robustness can be exploited in prolonged reactions, lowering the cost of the procedure. Moreover, they offer the possibility to perform the reaction at elevated temperatures, which in turn have many advantages, such as high solubility of the substrates and products, and low contamination risk.

All reactions were monitored for 72 h, in order to obtain a complete time profile of the reaction. The reactions were performed with different starting concentrations of acceptors, stevioside and rebA, in order to increase the conversion yields. Moreover, the reactions were also performed using low-cost industrial byproducts as donor sugars, acid whey as a source of lactose for *Tt*bGal1, and hydrolyzed microcrystalline cellulose (Avicel) as COS donor for *Mt*Bgl3a. Finally, the products obtained in all reaction conditions were detected by HILIC-HRMS analysis, confirming the successful transglycosylation of stevioside and RebA in all the above-mentioned reactions.

Regarding the transglycosylation performed by *Tt*bGal1, the conversion yields obtained were significantly higher than our previous results with the same enzyme, regarding the transglycosylation of lactose toward the production of GOS, which reached almost 20% (Zerva et al., 2021). In the case of stevioside modification, the maximum conversion yield is reached quickly, and consequently the products formed are probably hydrolyzed by the enzyme, similarly to the hydrolysis of the GOS produced by the same enzyme (Zerva et al., 2021). Interestingly, this was not observed in the case of rebA modification, since the conversion yield for this substrate kept increasing throughout the course of the reaction.

β-Galactosidases were previously used for the deglycosylation of stevioside to yield less glycosylated products. For example, β-galactosidase from *Klyveromyces lactis* was used for the production of steviolbioside (Chen et al., 2016) and from *Aspergillus* sp. (Wan et al., 2012), and *Thermus thermophilus* (Nguyen et al., 2014) for the production of rubusoside, since these enzymes were found active against the ester bond and the β-1,2 glycosidic linkage of stevioside, respectively. However, *Tt*bGal1 was not able to hydrolyze the above-mentioned bonds, since no hydrolysis products were detected when the enzyme was incubated with either stevioside or RebA in the absence of donor, and therefore it was shown to be an ideal catalyst for the transgalactosylation of both stevioside and RebA. On the other hand, a β-galactosidase from *Sulfolobus* sp., was found to both hydrolyze stevioside toward the production of steviol, and, with the addition of lactose as donor, transgalactosylate stevioside reaching up to 87.3% stevioside conversion (Wan and Xia, 2015).

β-Glucosidases also have been used widely for the hydrolysis of stevioside to less glycosylated products. Microbial β-glucosidases have been described in the literature, with the ability to cleave the β-1,2 glycosidic bond (Wang et al., 2015; Nguyen et al., 2018; Lan et al., 2019). In the present work, no hydrolysis products were detected when *Mt*Bgl3a was incubated with either stevioside or RebA.

Regarding *Mt*Bgl3a-mediated transglycosylation of steviol glycosides, the conversion yields obtained were similar to those obtained by *Tt*bGal1. The hydrolysis of the transglycosylation products was less prominent in this case, therefore, the products were shown to be more stable than in the case of *Tt*bGal1.

Aside from the transglycosylation of steviol glycosides, and the simultaneous hydrolysis of their respective donor sugars, both hydrolases were also found to transglycosylate their donors, lactose and cellobiose, to the respective oligosaccharides. The transgalactosylating activity of *Tt*bGal1, with lactose as the donor, was demonstrated in our previous study (Zerva et al., 2021). On the other hand, *Mt*Bgl3a was also shown to be capable of synthetic reactions, with cellobiose as the donor, but with methanol as the acceptor (Karnaouri et al., 2013). The presence of both COS and GOS in the final preparation can be beneficial for consumer health, since they are potent prebiotics (Torres et al., 2010; Kido et al., 2019).

As mentioned earlier, many different enzyme activities have been used in previous studies for the modification of steviol glycosides. Many studies report the transglycosylation with CGtases, with starch or other sugars as donors. Product yields exceed 80% with the use of such systems (Jaitak et al., 2009; Li et al., 2013; Lu and Xia, 2014). However, as mentioned earlier, the products formed from CGtases are mainly α-1,4- glycosylated, and therefore they can be hydrolyzed by human saliva amylases, resulting in a product with higher caloric content. On the other hand, glucansucrases are more promising enzymes in the modification of steviol glycosides, since they form a-1,6 and 1,3- glycosidic bonds, which are not hydrolyzed by human enzymes. Moreover, they can use low-cost donors, such as sucrose, reaching product yields over 90% (Ko et al., 2016; Devlamynck et al., 2019; Son et al., 2020). Regarding plant-derived UDP-glucosyltransferase- mediated bioconversion, the regeneration of UDP sugar donors with sucrose synthase is necessary, but yields over 70% can be obtained (Wang et al., 2016; Chen et al., 2021, 2020, 2018). Although all the above reported yields are undoubtedly higher than those reported in the present work, the glycosyl hydrolases used offer the advantage of forming β-1,4 glycosidic bonds, which cannot be broken by human digestive enzymes. One exception is human β-galactosidase, which can possibly liberate a β-1,4-bonded galactose from the modified steviol glycoside. In any case, regarding the organoleptic properties of the final product, sensory data are needed to determine the role of the introduced β-1,4 glycosidic bonds.

Another advantage of the enzyme systems proposed here, is the possibility of exploiting low-cost industrial byproducts, as shown by our results. Specifically, for β-galactosidase, the use of acid whey as sugar donor resulted in similar yields with pure lactose. The development an enzymatic process for stevioside modification with acid whey as the donor, could be implicated on-site in already existing strained yogurt production plants, simultaneously valorizing the liquid byproducts, and producing a high-quality non-caloric sweetener as an additive to the dairy products produced on the same plant. The ultimate goal of the design and implementation of such bioprocesses is the compliance of existing plants and production lines with the principles of circular economy.

Regarding the *Mt*Bgl3a – mediated bioconversion of steviol glycosides with hydrolyzed cellulose as sugar donors, the yields obtained were significantly lower than those with pure cellobiose solutions. However, in the present work, the realization of such a process was demonstrated, although extensive optimization is needed. Recently, a few studies report the development of efficient enzymatic cocktails for the production of COS from lignocellulosic biomass (Karnaouri et al., 2018, 2019). Similar enzyme systems, combined with *Mt*Bgl3a, could be exploited for the efficient modification of steviol glycosides from low-cost agroindustrial byproducts, consistent with the biorefinery concept.

## Conclusion

In the present work, a β-galactosidase *Tt*bGal1 and a β-glucosidase, *Mt*Bgl3a were applied in the transglycosylation of stevioside and rebaudioside A. Both enzymes proved effective in the tested reactions, resulting in very satisfactory conversion yields, which in most cases exceeded 30%. Moreover, both bioconversion reactions were performed with low-cost industrial byproducts as sugar donors, with very promising yields. The identification of the products by HILIC-HRMS identified the production of mono- and di-glycosylated products, proving the effectiveness of both enzymes in the reported reactions. Overall, the proposed transglycosylation systems offer a possibly feasible alternative in the biocatalytic modification of steviol glycosides, for the improvement of their taste profile. The simultaneous valorization of industrial byproducts, such as acid whey and cellulose hydrolyzate, could significantly lower the cost of the process, and offer an attractive alternative for the improvement of the organoleptic properties of stevia extracts, in agreement with the concept of circular economy.

## Data Availability Statement

The original contributions presented in the study are included in the article/Supplementary Material, further inquiries can be directed to the corresponding author/s.

## Author Contributions

AZ designed and performed experiments, analyzed data and wrote the original draft, with input from all authors. KC and AK performed experiments and analyses. NT supervised and designed experiments. ET designed and supervised the study and wrote the manuscript. All authors read and approved the final version of the manuscript.

## Conflict of Interest

The authors declare that the research was conducted in the absence of any commercial or financial relationships that could be construed as a potential conflict of interest.
