# Supplementary material for: β-Glucosidase and β-Galactosidase-Mediated Transglycosylation of Steviol Glycosides Utilizing Industrial Byproducts
Source: Front Bioeng Biotechnol. 2021 Jun 9;9:685099. doi: 10.3389/fbioe.2021.685099 (PMC8220073; doi:10.3389/fbioe.2021.685099)
Supplement: Supplementary file 1 [file Data_Sheet_1.DOCX]

Supplementary Material


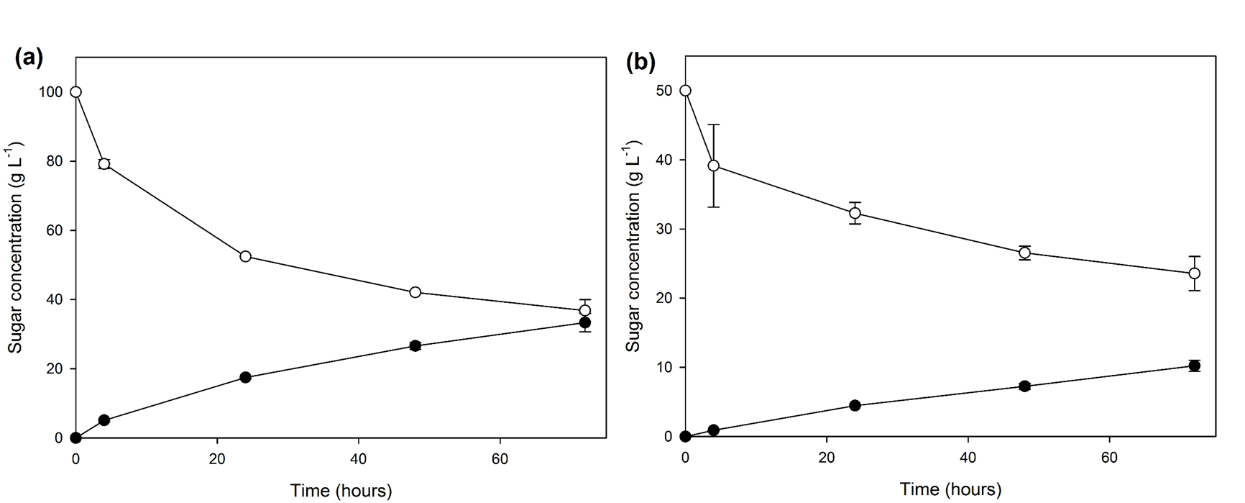


**Supplementary Figure 1.** Donor sugars conversion during transglycosylation of RebA by *Tt*bGal1 (a) and *Mt*BGL3a (b). White circles: disaccharide, black circles: monosaccharide.


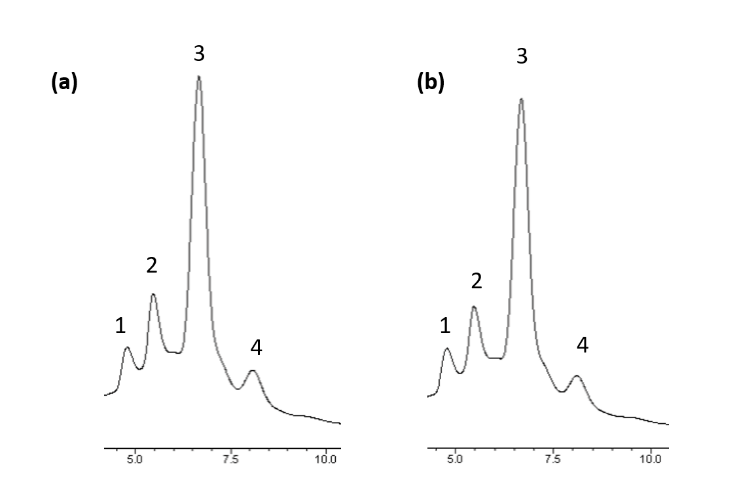


**Supplementary Figure 2.** HPLC chromatograms of the sugar composition of the transglycosylation reaction of RebA from *Tt*bGal1 (a) and *Mt*bgl3a (b), after 24 h. Peak 1: RebA, peak 2: monosaccharide, peak 3: disaccharide, peak 4: trisaccharide.


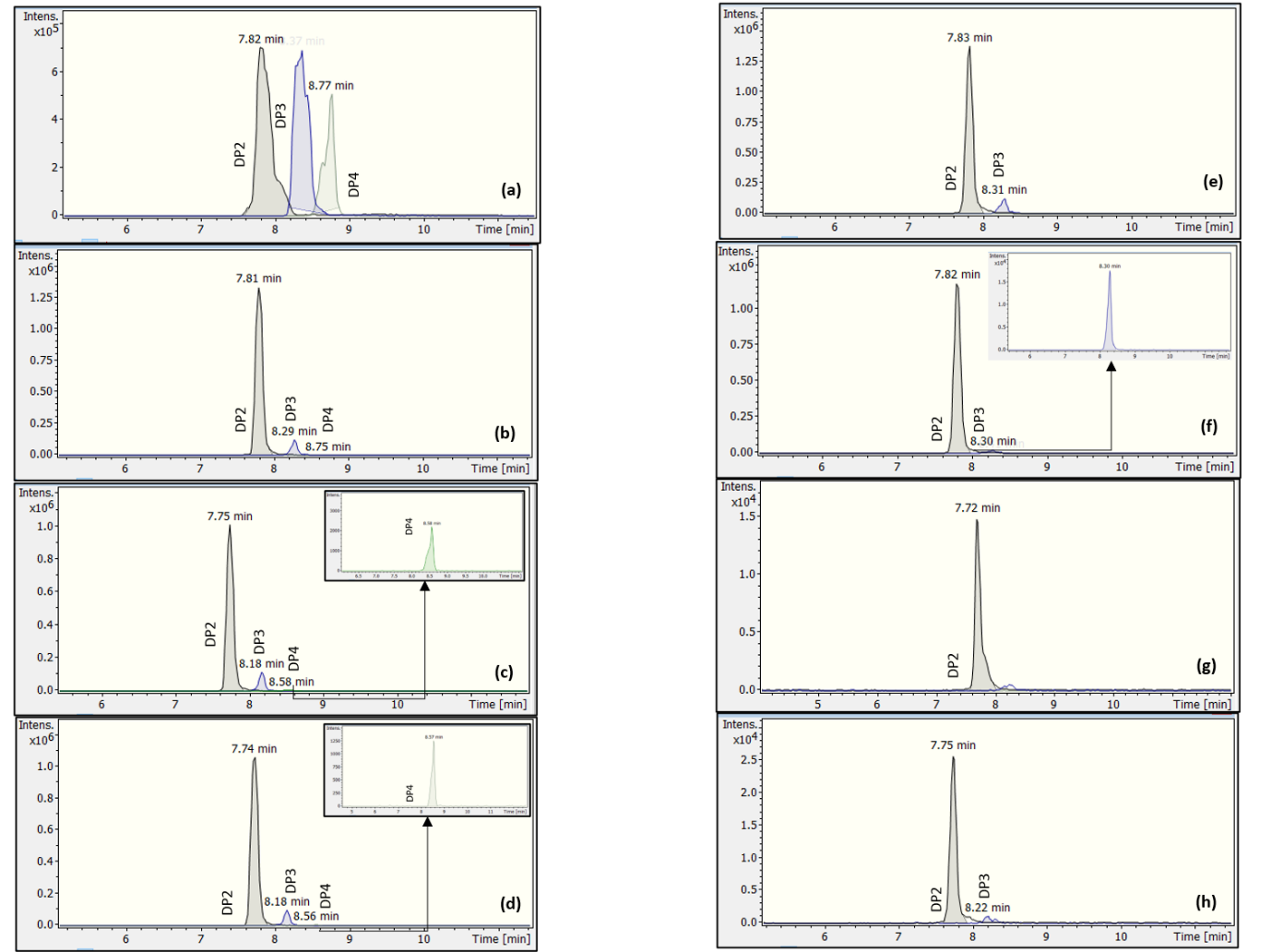


**Supplementary Figure 3.** Oligosacchrarides distribution due to side- reactions in samples: (a) *Tt*bGal1 – mediated transglycosylation of stevioside with lactose donor (b) *Tt*bGal1 – mediated transglycosylation of RebA with lactose donor (c) *Mt*BLG3a – mediated transglycosylation of stevioside with cellobiose donor (d) *Mt*BLG3a – mediated transglycosylation of RebA with cellobiose donor (e) *Tt*bGal1 – mediated transglycosylation of stevioside with acid whey donor (f) *Tt*bGal1 – mediated transglycosylation of RebA with acid whey donor (g) *Mt*BLG3a – mediated transglycosylation of stevioside with hydrolyzed cellulose donor (h) *Mt*BLG3a – mediated transglycosylation of RebA with hydrolyzed cellulose donor. DP: Degree of polymerization.


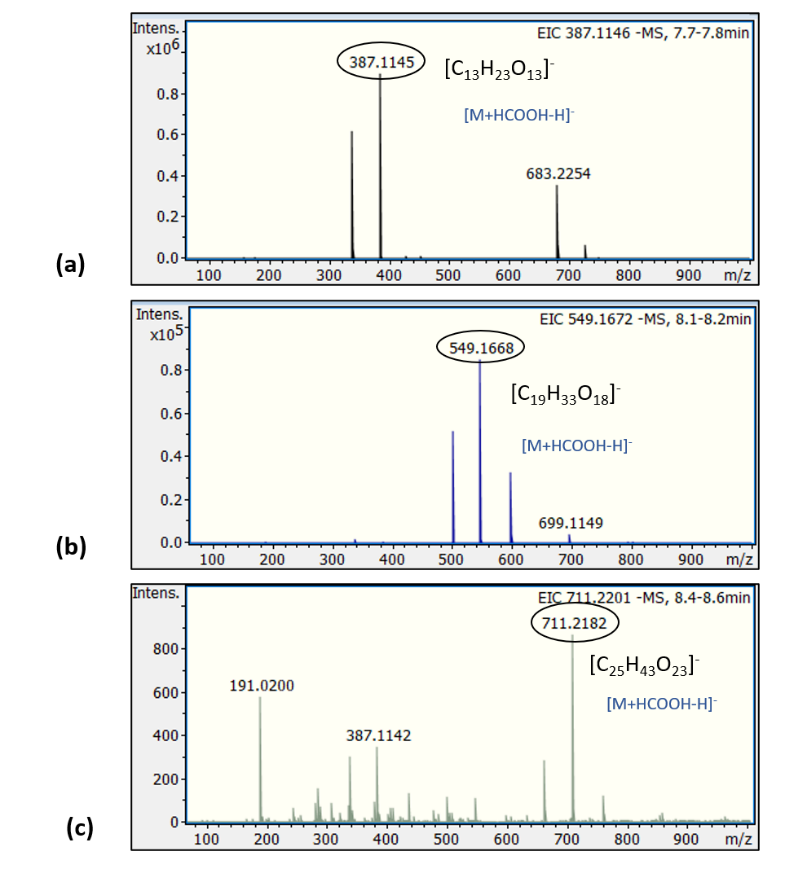


**Supplementary Figure 4.** MS spectra of cello-oligosacchrarides: (a) Cellobiose (C_12_H_22_O_11_), (b) Cellotriose (C_18_H_32_O_16_), (c) Cellotetraose (C_24_H_42_O_21_)
